# Impulsivity in ADHD and Borderline Personality Disorder: A Systematic Review of Gray and White Matter Variations

**DOI:** 10.3390/jcm13226906

**Published:** 2024-11-16

**Authors:** Łukasz Franczak, Piotr Podwalski, Patryk Wysocki, Bartosz Dawidowski, Adam Jędrzejewski, Marcin Jabłoński, Jerzy Samochowiec

**Affiliations:** 1 Department of Psychiatry, Pomeranian Medical University, Broniewskiego 26 Street, 71-460 Szczecin, Poland; lukasz.franczak@pum.edu.pl (Ł.F.); patryk.wysocki@pum.edu.pl (P.W.); bartosz.dawidowkski@pum.edu.pl (B.D.); marcin.jablonski@pum.edu.pl (M.J.); jerzy.samochowiec@pum.edu.pl (J.S.); 2 Independent Clinical Psychology Unit, Pomeranian Medical University, Broniewskiego 26 Street, 71-460 Szczecin, Poland; adam.jedrzejewski@pum.edu.pl

**Keywords:** borderline personality disorder, white matter, gray matter, impulsivity, attention deficit hyperactivity disorder, diffusion tensor imaging (DTI), emotional lability, magnetic resonance imaging

## Abstract

**Introduction**: Impulsivity is one of the overlapping symptoms common to borderline personality disorder (BPD) and attention deficit hyperactivity disorder (ADHD), but the neurobiological basis of these disorders remains uncertain. This systematic review aims to identify abnormalities in the gray and white matter associated with impulsivity in BPD and ADHD. **Methods**: We conducted a systematic search of the PubMed, Embase, and SCOPUS databases, adhering to PRISMA guidelines. Studies that investigated gray and white matter alterations in BPD or ADHD populations and their relationship with impulsivity were included. We reviewed information from 23 studies involving 992 participants, which included findings from structural MRI and DTI. **Results**: The review identified various nonhomogeneous changes associated with impulsivity in BPD and ADHD. BPD was mainly associated with abnormalities in the prefrontal cortex (PFC) and limbic areas, which correlated negatively with impulsivity. In contrast, impulsivity associated with ADHD was associated with structural changes in the caudate nucleus and frontal–striatal pathways. Despite the overlapping symptoms of impulsivity, the neurobiological mechanisms appeared to differ between the two disorders. **Conclusions**: These findings emphasize the distinct neurostructural correlates of impulsivity in BPD and ADHD. While both disorders show impulsivity as one of their main symptoms, the fundamental brain structures associated with this trait are different. BPD is primarily associated with abnormalities in the prefrontal cortex and limbic system, whereas the alterations seen in ADHD tend to focus on the caudate nucleus and frontostriatal pathways. Further research is needed to clarify these differences and their implications for treatment.

## 1. Introduction

Both borderline personality disorder (BPD) and attention deficit hyperactivity disorder (ADHD) are psychiatric disorders that strongly affect a patient’s global functioning [1]. Although their clinical presentation differs, there is a spectrum of symptoms that overlap in both of these disorders. Impulsivity, emotional dysregulation, and interpersonal impairment are the ones that make the differential diagnosis difficult [2]. Certain standardized psychometric scales can objectively assess impulsivity, an overlying symptom between the two disorders. 

BPD is a mental disorder that is characterized by unstable moods, impulsive behavior, and an unstable self-image. This disorder can lead to issues with relationships, family, and work life, as well as long-term planning [3]. It can also lead to tendencies toward self-harm or suicidal behavior, with up to 10% of BPD patients committing suicide [4]. Other symptoms of BPD include drug or alcohol abuse, overspending, binge eating, or risky sexual behavior [5]. The prevalence of BPD in the general population is approximately 1%. The prevalence of BPD is substantially higher in clinical settings—around 12% in the outpatient psychiatric population and 22% among inpatients [6]. The etiology of BPD is widely believed to be multifactorial, mentioning both environmental and biological factors [7]. Several recent reports have consequently valued the heritability of BPD at about 46% [8].

A number of meta-analyses show that the left and right hippocampus and the left and right amygdala both have smaller gray matter volumes (GMVs) [9,10,11,12]. However, more recent studies have not confirmed these findings [13,14,15]. Compared to the control group, people with BPD had less dense cortical gray matter. This was especially noticeable in the dorsolateral frontal cortex (DFC), orbitofrontal cortex (OFC), anterior and posterior cingulum, right parietal lobe, temporal lobe (medial temporal cortex and fusiform gyrus), and visual cortex [16]. Furthermore, BPD patients show reduced GMV in the posterior cingulate gyrus (PCG) [17], the bilateral anterior cingulate gyrus (ACC), right middle temporal gyrus (MTG), left middle occipital gyrus (MOG), left inferior frontal gyrus (IFG), left middle frontal gyrus (MFG), and right insula compared with healthy controls (HC) [14,15]. These findings provided proof of structural connectivity abnormalities in BPD patients [18]. A review of many diffusion tensor imaging (DTI) studies found that there was a statistically significant drop in fractional anisotropy (FA) in the genu and rostral areas of the corpus callosum (CC), as well as in the left and right prefrontal white matter fasciculi, in people with BPD compared to healthy controls [19].

ADHD is characterized by recurrent patterns of impulsivity and emotional dysregulation, which can be observed in BPD [20,21]. Furthermore, compared to the prevalence of ADHD in children, which ranged from 10.5 to 11.5% [22], the prevalence of ADHD in adults was 6.76% [23]. Research has proven that patients with ADHD are more likely to receive diagnoses for other psychiatric comorbidities, specifically substance abuse, mood and anxiety disorders, and personality disorders [24]. The evaluation of the comorbidity ranged from 32.4% to 38.1% of the examined BPD population [25,26].

The complicated biological underpinnings of ADHD include immunological and genetic variables. Studies show that there is a significant hereditary component to ADHD. The likelihood of acquiring ADHD is higher in siblings of individuals with the illness. Researchers believe that 40–80% of ADHD cases are heritable [27]. There is extensive literature documenting associations between ADHD and a wide range of putative environmental risks, such as stress, maternal prenatal stress, fetal infections, and exposure to toxic substances such as alcohol, nicotine, lead, or pesticides [28].

Neuroimaging studies have identified broad structural alterations in several brain regions linked to ADHD. Structural magnetic resonance imaging (MRI) typically reveals a general decrease in brain size, which persists into puberty [29]. ADHD is associated with regional reductions in the volumes of the right caudate, total and right cerebral volumes, and the volume of the splenium of the CC [30]. Meta-analyses and mega-analyses have reviewed the structural MRI studies conducted over the past two decades, revealing consistently replicated alterations in the basal ganglia and several other subcortical areas. The voxel-based meta-analysis of diffusion studies found that people with ADHD had both higher and lower FA in different parts of the brain, mostly in the cingulum, the CC, and the left inferior fronto–occipital fasciculus (IFOF) [31,32,33,34]. Compared to children who typically develop, children with ADHD have demonstrated alterations in their prefrontal and striatal connections as well as decreased frontal–accumbal white matter connectivity [35].

Impulsivity can be considered a personality trait defined by a tendency to act on immediate urges, either before consideration of possible negative consequences or despite consideration of likely negative consequences [36]. Impulsivity can show up in a number of ways, including emotional reactivity, spontaneous statements, and trouble with delaying satisfaction [37,38]. Impulsivity is a trait found in many psychiatric disorders, as summarized in Table 1 [39,40].

According to contemporary classifications, impulsivity is considered a key pathological concept in the diagnostic criteria of BPD and ADHD. A meta-analysis revealed a negative correlation between the GMV and the trait of impulsivity [41]. Previous studies have linked the same regions of PFC to BPD and ADHD symptomatology.

However, the studies indicate that certain regions of the brain are associated with the impulsivity trait [41]. These regions are not completely in the same location as parts of the brain that are changed in people with ADHD and BPD [42], which may suggest a different cause for the impulsivity that is a feature of these disorders. This study seeks to investigate if the disruption of gray matter and white matter in the ADHD and BPD populations reflects any abnormalities associated with impulsiveness.

## 2. Materials and Methods

We conducted a systematic evaluation of research on white matter and gray matter abnormalities in ADHD and BPD after pre-registration in PROSPERO (CRD42023424200). The authors undertook a systematic search of three electronic databases in accordance with the PRISMA 2020 guidelines for reporting systematic reviews [43].

### 2.1. Literature Search

The following search terms, in various combinations (including acronyms), were used in the MEDLINE, Embase, and SCOPUS databases and were initially carried out in January 2024. An updated search was carried out in July 2024 and did not lead to any new articles being added to the review. A range of terminology, primary keywords restricted to English-language and human-only studies, and medical subject headings (MeSH) were applied. The keywords we used according to the PICO framework were “borderline personality disorder”, “white matter”, “gray matter”, “impulsivity”, “attention deficit hyperactivity disorder”, “diffusion tensor imaging”, “emotional lability”, and “magnetic resonance imaging”.

### 2.2. Inclusion and Exclusion Criteria

Original research articles published before January 2024 that fulfilled the following requirements were included: (1) The study sample consisted of people with BPD or ADHD; (2) the diagnosis of BPD or ADHD was conducted using one of the DSM-3/DSM-4/DSM-5 or ICD-10 criteria; (3) DTI or MRI was the neuroimaging technique used; (4) impulsivity was measured; (5) healthy controls (HC) were included; and (6) the publication was written in English.

The following were the exclusion criteria: (1) non-original data (case studies, conference abstracts, systematic or non-systematic reviews, etc.); (2) non-ADHD or non-BPD individuals (or studies using a combination of samples of patients with a different diagnosis and ADHD or BPD patients); (3) different neuroimaging techniques (no assessment of white matter or gray matter); (4) studies including individuals who may have potential cerebellar lesions from which the observed disorders will result; and (5) languages other than English.

### 2.3. Data Extraction

Two independent researchers (ŁF and PP) initially chose records for inclusion. Consensus was used to resolve any differences. The eligibility of search results was verified twice: first by title and abstract, and then by full text. Every study that was excluded had its reasons for exclusion documented. We carried out an additional manual search of the references in the papers that we downloaded from the databases. The primary researcher utilized a Microsoft Excel spreadsheet to gather important data from each study.

The following data were extracted from the included studies: authors, year of publication, methodology, and socio-economic and medical indicators that were used in the original analyses (sample size and its characteristics, i.e., age range, gender distribution, substance use, medication status), and any coefficients/descriptive statistics required in the analysis of the relationship between white matter and gray matter alterations and symptom severity and level of functioning.

On 19 January 2024, a preliminary search produced 213 records, after removing duplicates, entries not in English, books, theses, dissertations, and non-peer-reviewed publications. We carried out an initial search on 9 February 2024. An additional 88 records were found by this search, most of which came from citation searches of previous systematic reviews and meta-analyses. An updated search was carried out in July 2024 and did not lead to any new articles being added to the review. Two members of the review team separately inspected each record during the abstract review. When there was disagreement among the authors regarding the study to be done, a unanimous decision was reached. Articles were kept only if they satisfied the previously stated inclusion and exclusion requirements (see Section 2.2). Only eligible articles, as per the designated inclusion and exclusion criteria, were retained, with 23 articles identified as appropriate to enter the review.

### 2.4. Risk of Bias Assessment

All included studies were evaluated for quality and bias risk. A revised version of the Effective Public Health Practice Project (EPHPP) quality assessment instrument was utilized [44,45]. Studies were evaluated for potential selection bias, the suitability and rigor of the study design, the validity of social cognition awareness and function tasks, neuroimaging methodologies, and the appropriateness of statistical analyses employed. Each subsection received a quality rating of “high”, “moderate”, or “low”, which informed the overall quality assessment (high = no low ratings in any subsection, moderate = one low rating, low = more than two low ratings) (Supplementary Table S1).

## 3. Results

### 3.1. Study Selection

**Figure 1:**
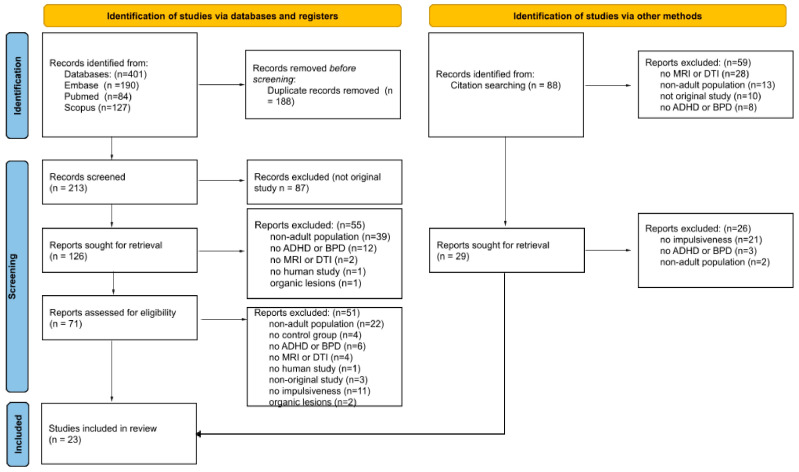
PRISMA flowchart for the inclusion of studies.

We identified 401 studies across MEDLINE, Embase, and SCOPUS databases, 188 of which were duplicates, and identified 88 records via citation searching (Figure 1). A total of 200 studies were excluded at the title-abstract level, and 76 were excluded after examining the full texts. Table 2 and Table 3 display the main characteristics of the 23 studies we ultimately included. Clinical centers in Europe, the U.S., and Asia conducted a total of 16 studies. The included studies were published between 2005 and 2023. The sixteen qualified articles assessed patients with a diagnosis of BPD (Table 2), six studies assessed individuals who matched criteria for ADHD (Table 3), and one study qualified patients who met criteria for both disorders. Of the eligible studies that assessed patients with a diagnosis of BPD, twelve examined gray matter changes and six examined white matter changes. Of the studies that assessed patients with a diagnosis of ADHD, two assessed white matter and six assessed white matter changes. The sample sizes of the twenty-three studies varied from seven to one hundred and seven ADHD/BPD individuals, for a total of nine hundred and ninety-two participants. The mean age of patients was 29.96 years; all the participants were adults; and all except for eight articles included both male and female participants.

### 3.2. Intervention Characteristics

All the studies used various magnetic resonance imaging sequences. Fourteen studies used structural MRI, eight studies used DTI, one study used diffusion spectrum imaging (DSI), and one study used both structural MRI and DTI sequences. Studies compared specific morphometric variables of the subjects’ cerebrum with a control group. Investigations using structural MRI used parameters such as GMV, white matter volume (WMV), gray matter concentration (GMC), cortical thickness (Cth), local Gyrification Index (LGI), hemispheric asymmetry, and volumes of specific brain areas. Recent studies, using a broad-dimensional approach, have linked changes in brain function and structure to higher-order psychopathological factors in both adolescents and adults, using the aforementioned parameters among others [69,70,71,72,73]. DTI enables analysis of the structural connectivity in neurodegenerative diseases such as amyotrophic lateral sclerosis (ALS) [74,75], Alzheimer’s disease [76], and Parkinsonism [77,78]. Successful attempts have also been made to associate the WM abnormalities observed in DTI with schizophrenia [79] and ultra-high-risk psychosis state patients [80]. DTI can use metrics such as fractional anisotropy (FA), mean diffusivity (MD), axial diffusivity (AD), and radial diffusivity (RD) to measure the integrity of white matter tracts in vivo [81,82]. FA serves as a concise indicator of microstructural integrity. FA is less specific to the type of change, but it is highly sensitive to microstructural changes. While AD typically exhibits variability, MD functions as an inverse measure of membrane density. The DSI study measured mean generalized fractional anisotropy (mGFA). DSI can be used to investigate the characteristics of WM microstructures. The GFA diffusion anisotropy indexes used in DSI are the counterparts of the FA indexes used in DTI [83]. The approaches taken by these two techniques to deal with crossing fibers, however, differ. Whereas the DTI method indicates a direction between the crossing fibers, DSI can provide the directions of the maxima of the average propagator corresponding to the underlying crossing fiber directions [84].

Based on our quality assessment, one study was classified as low quality, thirteen as moderate quality, and nine as high quality. Nine studies were assessed as high quality, as they did not receive low ratings in any quality domains. Thirteen were classified as moderate, attributed to one inadequate rating in the neuroimaging methodology criteria, selection bias, and two small study samples along with the analysis. One study received a lower quality rating due to inadequate evaluations in the criteria for selection, neuroimaging methodology, and analysis (Supplementary Table S1).

### 3.3. Intervention Measurements

The level of impulsivity was measured with the Barratt Impulsiveness Scale (BIS-11), the ADHD Rating Scale-IV (ASRS), the Test of Variables of Attention (TOVA), Eysenck’s Impulsivity Inventory (IVE), and the Revised Diagnostic Interview for Borderlines (DIB-R). One study utilized multiple scales from the list above. The BIS-11 consists of six main factors and three second-order factors. The three second-order factors were labeled Attention Impulsivity, Motor Impulsivity, and Non-Planning Impulsivity. The BIS-11 total score is an internally consistent measure of impulsivity and has potential clinical utility for measuring impulsivity among selected patient populations [85,86]. The ASRS is a self-report screening scale for ADHD in adults developed by the World Health Organization (WHO). The ASRS contains 18 questions on the frequency of recent ADHD symptoms in adults with ADHD. Each ASRS symptom measure was significantly associated with comparable scores on clinical symptoms, including impulsivity [87]. The CAADID is a structured interview that assists in the process of diagnosing adult ADHD. The interview consists of two independent parts, Part I and Part II. The Patient History Questionnaire, or Part I, can be used as a self-report questionnaire or as a clinical interview. It asks about the client’s medical background, how their attention issues have developed over time, related risk factors, and comorbidity issues. Part II presents the Diagnostic Criteria Interview, which incorporates impulsivity [88]. The TOVA is a neuropsychological assessment that measures a person’s attention while screening for ADHD. When the subject responds to a “non-target”, it is noted as an error of commission or impulsive [89]. The IVE was created to evaluate the impulsive, adventurous, and empathic personality traits. It is used as a measure of risk preferences as well as the personality constructs themselves because it is assumed that impulsivity and venturesomeness contribute to risk preferences [90]. The DIB-R measures four major aspects of BPD: affect, cognition, impulse action patterns, and interpersonal relationships [91].

### 3.4. Relationship Between Impulsivity and Neuroimaging Findings in BPD

Sixteen studies focused on the association of impulsivity and the neurobiological substrate associated with BPD in the control group (Table 2). Twelve of them examined gray matter changes, and six examined white matter changes. The sample sizes of the sixteen studies ranged from seven to sixty-one BPD individuals, for a total of four hundred and eighty-four participants. The mean age of patients was 29.26 years; all participants were adults; eight articles included only the female population. BPD patients reported a range of comorbid psychiatric disorders, including major depressive disorder (MDD), substance use disorder (SUD), post-traumatic stress disorder (PTSD), anxiety disorders, eating disorders, adjustment disorders, other personality disorders, and somatoform disorders. One study included patients with comorbid acute psychotic disorder [56]; another study included patients with a diagnosis of schizophrenia [46]. Most publications did not focus on the impact of comorbidities on a variety of clinical and research issues related to the neuroimaging of patients with BPD. One study compared patients with OUD, BPD, CUD, and SZ with a control group [46], while two others compared patients with MDD and BPD with a control group [48,50]. From both of these papers in the review, we only extracted data comparing patients with a diagnosis of BPD versus HC. The study groups exhibited heterogeneity in terms of medications taken; five studies included patients who were not taking any medications at the time of the study [51,54,55,57,62], while one study exclusively recruited drug-naive patients [61]. In the remaining studies, the study groups consisted of people who were taking psychopharmacology. In three studies, groups consisted of both patients who were taking medication and patients who were functioning without psychotropic medication [46,53,56]. When analyzing the papers according to the impulsivity scales used, one paper used the IVE [56], one paper used the DIB-R [60], and the rest of the studies used the BIS-11.

Twelve papers were based on structural MRI examinations; six of these examined the relationship between impulsivity and GMV, one investigated the correlation between impulsivity and Cth, another examined the relationship between impulsivity and WMV, and one focused on the relationship between impulsivity and GMC. The authors of one study examined the relationship between impulsivity and LGI, another examined the relationship between impulsivity and hemisphere asymmetry, and the final study investigated the relationship between impulsivity and the hippocampal and caudate nucleus volumes in patients in the study groups.

Schaub et al. showed a negative relationship between GMV in the left IFG volume and the BIS-11 motor factor, but not with the attention and non-planning factors [46]. In a different study, Völlm et al. found that impulsivity was negatively linked to GMV in the OFC, MFG, precentral and postcentral gyrus, temporal pole, inferior and superior parietal cortex [54]. It was also shown by Sampedro et al. that impulsivity was negatively related to GMV in the middle and inferior prefrontal areas of the left hemisphere, while it was positively related to GMV in the PCC, precuneus, and parahippocampal areas [47]. Sala et al. demonstrated a negative correlation between bilateral dorsolateral prefrontal cortex (DLPFC) GMV and impulsivity [53]. Sanpedro et al. also found impulsivity was negatively correlated with Cth in the caudal and middle frontal and precentral areas [47]. Depping et al. examined the link between LGI and impulsivity using a different method and found that the LGI of the medial orbitofrontal gyrus and of the rostral MFG were negatively related to impulsivity [48].

Other papers focused their studies on structures other than the frontal lobe. Depping et al. proved a negative correlation between the GMV of the hippocampus, parahippocampus, amygdala, and impulsivity [50]. In contrast, Kuhlmann et al. showed no correlation between GMV of the hippocampus, amygdala, ACC, hypothalamus, and impulsivity [51]. These findings were consistent with another paper by Zetzsche et al., which showed no correlation between impulsivity and hippocampal GMV [56]. Moreover, Soloff et al., examining the relationship between GMC and impulsivity, found no correlation between impulsivity and GMC in any brain region studied [55]. However, O’Neill et al., studying hippocampus and caudate nucleus volume, demonstrated no significant correlation between hippocampal volumes and impulsivity and a negative correlation between right caudate volume and impulsivity [53]. Zhou et al. achieved interesting results, showing no significant correlation between total impulsivity scores and left-right hemisphere asymmetry in ACC Cth and the anterior insula (AI) Cth. However, the authors did find a positive correlation between the asymmetry in the ACC Cth and the score on the BIS attention subscale, as well as a positive correlation between the left-right hemisphere asymmetries in the AI GMV and the score of the BIS attention subscale [49]. In addition, Hazlett et al. examined Brodmann areas and their association with BPD symptomatology. The authors showed a negative correlation between left GMV in BA 25 and impulsivity, and a negative correlation between left GMV in BA 10 and impulsivity. Researchers studied the WM and found that there was a negative relationship between the right WMV in BA23 and impulsivity. They also found a positive relationship between the left and right WMV in BA44 and impulsivity, and a positive relationship between the WMV in BA47 and both impulsivity and irritability-assaultiveness [57]. The study describes changes such as reduced GMV and WMV in the cingulate region (anterior: BA 25; posterior: BA 23), smaller GMV in the inner prefrontal areas (BA 10), and increased WMV in the orbital area (BA 47) and DLPFC (BA 44).

Four papers were based on DTI examination. All articles examined the relationship between FA and impulsivity in patients in the study groups. Additionally, Salvador et al. examined the correlation between MD, global brain connectivity (GBC), amplitude of low-frequency fluctuations (ALFF), and impulsivity [60], and two studies examined RD [58,61]. All researchers found no correlation between the diffusion parameters and impulsivity. However, Gan et al. discovered a negative relationship between the genu and body of the CC and unplanned impulsivity, a positive relationship between the anterior thalamic radiation (ATR) and attention impulsivity, and a negative relationship between the fiber bundles passing through the fornix and positive intensity and motor impulsivity [61]. After multiple comparison adjustments, the effect diminished and was no longer statistically significant. We identified one study that tested the correlation between impulsivity and organic brain changes in a population of patients with BPD and ADHD comorbidity [59]. The authors examined patients’ brains using DTI. They studied FA and MD parameters in the corpus callosum. They found no correlation between FA or MD in any region of the CC and impulsivity in these patient groups.

### 3.5. Relationship Between Impulsivity and Neuroimaging Findings in ADHD

Six studies examined the connection between impulsivity and structural alterations in ADHD patients’ brains (Table 3). Only two of the studies examined gray matter changes, and six examined white matter changes. The six studies’ sample sizes ranged from 35 to 119 ADHD individuals, for a total of 453 participants. The mean age of patients was 32.98 years; all participants were adults; and all of the articles included male and female populations. A variety of comorbid mental illnesses, such as MDD, bipolar disorder (BP), SUD, anxiety disorder, BPD, and antisocial personality disorder (ASPD), were mentioned in ADHD patients. In most publications, the aim of the study and the research questions did not focus on comorbidities affecting a range of clinical and research issues in the neuroimaging of patients with ADHD. The medication regimens of the study groups varied; two of the studies [67,68] included patients who had never taken any medications. In the remaining studies, the study groups consisted of individuals taking psychopharmacology, while in one study, the study group included both medication-taking patients and drug-naive patients [63]. In one study, the patients underwent a 48 h medication washout period prior to an MRI [65]. When analyzing the papers according to the impulsivity scales used, one paper used CAADID [65], one paper used TOVA [67], and the remaining studies used the ASRS.

Two articles were based on GM examination [63,64]. Onnink et al. proved a negative correlation between right caudate volume and hyperactivity/impulsivity and stated that structural differences are less pronounced in females than in males [63]. On the contrary, Wolfers et al. found no correlation between Cth, GMV, and impulsivity in the ADHD group [64].

The DTI exam served as the foundation for four papers. Every article examined the connection between the study group patients’ impulsivity and FA. Furthermore, two records examined the relationship between MD and impulsivity [64,67], and another record examined MD, AD, and RD [66]. There was no association between impulsivity and the neuroimages that were obtained, according to two studies [64,66]. However, Luo et al. demonstrated a negative correlation between hyperactivity/impulsivity and FA of the left caudate-parietal WM fiber tract [65]. Konrad et al. demonstrated a positive correlation between MD bilaterally in the lingual gyrus and impulsivity and a negative correlation between FA and impulsivity in the right frontobasal WM, including parts of the right uncinate fasciculus (UF) and the right ATR [67].

One paper was based on the DSI examination [68]. It examined the relationship between mGFA and impulsivity in patients in the study groups. The authors demonstrated a negative correlation between the mGFA values of the right superior longitudinal fasciculus (SLF) and the right frontostriatal tract from the DLPFC and symptoms of hyperactivity and impulsivity.

### 3.6. Relationship Between Impulsivity and Neuroimaging Findings in ADHD and BPD

In conclusion, the structural findings in BPD always show that impulsivity is negatively correlated with structures in the PFC, ACC, and PCC. Some studies also indicate a negative correlation between the GMV of other limbic structures and impulsivity. In studies based on neuroimaging in the DTI modality, most authors found no association between the parameters studied and impulsivity. Authors investigating changes in patients with a diagnosis of ADHD tended to focus on neuroimaging in the DTI sequence, most of them failing to find a link between the parameters investigated and impulsivity. A study examining structural changes revealed a negative correlation between the GMV of the caudate nucleus and the semiology of impulsivity [63], a finding also confirmed in a study on BPD patients [47]. On the basis of these results, it is difficult to draw clear conclusions about the common neurobiological basis for impulsivity in BPD and ADHD.

## 4. Discussion

To our knowledge, this is the first comprehensive comparative analysis of the neural correlates of structural features associated with impulsivity in patients with BPD and ADHD. Based on the detailed results of the studies that were part of the review, we can see that people with BPD and ADHD both have abnormalities in the temporoparietal connections and in the frontostriatal circuits. Our findings provide a new insight into the neurobiological changes that may underlie impulsivity in these patient populations. Existing literature supports the alterations we identified, despite their inconsistent replication across studies. Despite the variability and lack of homogeneity among patients in different groups, our review highlights specific areas that may be responsible for the increased impulsivity characteristic of BPD patients. These findings could help elucidate the neurological basis of impulsivity in BPD and inform future research on the disorder’s nature, progression, and treatment. However, due to the limited availability of literature at the time of this review, we were unable to draw definitive conclusions about the neurobiological underpinnings of impulsivity in ADHD or broadly link these to the changes observed in BPD patients. Notably, we did identify a relationship between the GMV of the caudate nucleus and impulsivity in two studies on BPD and one study on ADHD. Nevertheless, identifying a common neural substrate for impulsivity in both ADHD and BPD remains elusive from a broader perspective.

Impulsivity in ADHD can be explained by impaired inhibitory control within a ‘top-down model’ in which malfunction of higher executive functions in the prefrontal cortex leads to difficulties in regulating impulsive reactions. The top-down model, in the context of cognitive science, describes how our goals, expectations, and prior knowledge influence our perception, behavior, and information processing. It is a way of understanding how the brain does not just passively receive sensory input but actively shapes and interprets that input based on what we already know and what we want to achieve [92,93]. A deficiency in top-down cognitive control, often associated with impulsivity, is characterized by actions that are poorly conceived, prematurely expressed, unduly risky, or inappropriate to the situation, often resulting in undesirable outcomes [94]. This condition exists at one end of a spectrum of compulsive and impulsive disorders [95]. According to the top-down model, PFC activity mediates the guiding influence of our goals, intentions, and past knowledge on our perception and behavior [96,97]. In the hippocampus, the PFC exerts top-down control over information processing by acting through a unique circuit motif: long-range GABAergic projections [98]. The PFC is specifically in charge of executive processes, such as inhibition of inappropriate behavior, planning, and decision-making [99,100]. A decreased ability to perform top-down control can result from decreased PFC activity [24], which can be caused by neurological conditions [101,102,103], psychiatric conditions such as ADHD or BPD [104,105,106,107], or stress [108,109]. Regarding the groups we examined, this hypothesis suggests that problems in some parts of the prefrontal cortex lead to weaker control over parts of the brain that handle emotions and rewards, such as the amygdala and striatum (which includes the caudate nucleus). In BPD, this impaired top-down modulation is thought to contribute to the increased emotional dysregulation, perceived emotional pain, and impulsive behavior characteristic of the disorder, driven by hyperactivity in limbic areas [110,111,112]. Similar deficits in prefrontal cortex functioning underlie impulsive actions in ADHD, as impaired top-down control fails to adequately suppress immediate reward-seeking behavior [113]. These prefrontal-subcortical neurobiological dysfunctions may be underlying the pathophysiology of impulsivity in both disorders.

In BPD, most of the negative correlations found in MRI referred to changes in the PFC, including IFG, OFC, and DLPFC [46,47,48,53,54]. Positive correlations were also found between impulsivity and GMC BA 44 and BA 47 (which refer to the pars opercularis of the inferior frontal lobe and the gyral region of the orbital part of the inferior frontal lobe) [57]. Further changes were related to the negative correlation of the GMV of the limbic system [50,52]. The study concluded an interesting relationship between impulsivity and asymmetry in the Cth and GMV of the ACC and AI. They showed an association only between changes in the asymmetry of these structures and the BIS attention score, with no correlation with the BIS total score [49]. O’Neil et al. showed completely different results for HC than for the BPD group, which may suggest a different neurobiological basis in patients in this group. HC showed a statistically significant negative correlation between both hippocampal tail volume and impulsivity and a negative correlation between right hippocampal tail volume and impulsivity. The BPD group did not confirm these correlations. Conversely, the BPD group observed a negative correlation between right caudate volume and impulsivity [52]. There is no direct link between the differences in symptoms and the changes seen in the DTI sequence according to most authors. However, one study found that impulsivity was negatively correlated with the FA of the genu and body of the CC, positively correlated with the RD of the ATR, and negatively correlated with the FA of the fiber bundles passing through the fornix. After multiple comparison corrections, the effect faded and was no longer statistically significant [61].

The IFG is an important region implicated in a variety of tasks, including language comprehension, speech production, semantic processing, fine motor control, interoceptive awareness, and emotion [114,115,116]. It is known for being the center for language production, or “Broca’s Area”, which can cause expressive forms of aphasia when injured [117,118,119]. Research suggests that the IFG plays a crucial role in cognitive processes related to motor inhibition [120,121], particularly in response inhibition in HC [122,123]. Various psychiatric disorders such as anorexia nervosa [124], obsessive-compulsive disorder (OCD) [125,126], BPD [13,42], ADHD [42,127], BP [128,129], MDD [129], and alcohol dependence [130] exhibit GMV reduction in IFG. Literature suggests that the effective application of inhibitory control over motor responses depends on the integrity of the left IFG. IFG can control trait impulsivity by focusing on the inhibitory response [131,132]. Considering the symptomatology of BPD, these findings may help to understand the underlying impulsivity and patient-reported ‘lack of emergency brake’. 

The OFC plays a key role in decision-making, referred to as impulsivity without planning [133]. According to Rolls et al., this area essentially reflects the processing of reward after goal-directed activity to steer adaptive behavior [134,135]. OFC lies at the interface of emotion and cognition. It plays a central role in the ability to make predictions about the likely consequences of potential actions so that we can make optimal decisions [136]. The findings show that in healthy subjects, GMV of OFC is inversely correlated with impulsivity [133,137,138,139], which is consistent with our findings. Furthermore, Depping et al. were the first to demonstrate that OFC hypogyrification was associated with impulsivity [48]. GMV reduction in OFC is seen in disorders like social anxiety [140], alcohol and drug dependence [141,142], ADHD [42,143], disinhibited eating and food addiction [144], and BPD [145]. Suicide attempts were associated with decreased lateral OFC thickness and decreased OFC volume [146,147,148,149]. Additionally, left lateral OFC volumes were associated with more lethal suicide attempts [148].

Dalwani et al. found a similar link between impulsivity and lower GMV in the DLPFC, which aligns with our findings [150]. Nonetheless, prior research by Gavazzi et al. demonstrated that the right MFG is a part of the inhibitory system and plays a role in the inhibitory control of executive processes [151]. This system is linked to behavioral manifestations of impulsivity [99], and impulsivity is elevated when top-down executive functions are compromised [152]. More precisely, reactive inhibitory control processes are the only ones in which MFG is engaged [151,153]. Impaired inhibitory control results in increased decision-making impulsivity when neural treatments are used in the appropriate MFG in healthy adults [154]. Functional neuroimaging studies on incarcerated adults with psychopathic traits linked hyperimpulsivity to increased functional connectivity between the left MFG and right OFC [155]. This suggests that there may be interactions in PFC regions that are associated with reward value judgment and behavioral planning, respectively. A GMV reduction in MFG was observed in disorders like anxious depression [156], alcohol and drug dependence [141,142], ADHD [42,143], disinhibited eating and food addiction [144], and BPD [145].

An interesting conclusion was reached by Zhou et al., which showed no significant correlation between total impulsivity scores and left-right hemispheric asymmetry in ACC Cth in BPD [49]. However, the authors did find a positive correlation between the asymmetry in the Cth of ACC and the score of the BIS attention subscale, as well as a positive correlation between the left-right hemisphere asymmetries in the AI GMV and the score of the BIS attention subscale. The study found no significant correlation between the total impulsivity scores and the left-right asymmetry of AI [49]. This is consistent with the findings of other researchers who have shown that increased impulsivity is also characterized by increased left hemisphere activity [157,158,159].

O’Neil and colleagues found no significant correlation between hippocampal volume and impulsivity in BPD. However, it proved a negative correlation between right caudate volume and impulsivity in this group. This aligns with the findings of Volmm et al. [54]. Overall caudate volume asymmetry has been linked to both motor and attentional forms of impulsivity, in line with some studies [160,161]. Innovative research on Rhesus monkeys also confirmed this model. Eldridge et al. provided evidence that the caudate nucleus contributes to the inhibition of motor impulses by demonstrating that unilateral suppression of the caudate nucleus is sufficient to cause an observable behavioral deficit, which manifests as an increase in impulsivity [162]. GMV reduction in the caudate nucleus is associated with disorders like ADHD [163], BP [164], autism spectrum disorder (ASD) [165], MDD [166], OCD [167], PTSD [168], and schizophrenia [169].

According to three meta-analyses, studies comparing BPD to HC have revealed notable volume reductions in limbic and paralimbic regions, most notably in the hippocampus and amygdala [9,10,11]. GMV reduction in amygdala and hippocampus was observed in disorders like ASD [170], PTSD [171], schizophrenia [172], BP [173], BPD [11], and MDD [174]. Patients’ propensity for impulsivity, aggression, and emotional reactivity may be associated with the temporal-limbic system. Specifically, there has been evidence of a decrease in the amygdala and hippocampal volumes in the temporal-limbic system. The aforementioned discoveries carry noteworthy therapeutic consequences, given that these structures play a role in modulating both violent conduct [175] and emotions [176]. Other researchers found a negative correlation with the amygdala [50,177,178]. The amygdala also made a distinction between BPD and MDD, indicating that a connection exists that goes beyond the conventional understanding of depression to explain the impulsivity and affective instability seen in BPD. Keeping negative emotions in check can affect how people with non-planning impulsivity see the goals of their actions, and the temporal pole is important for processing sensory inputs with the amygdala [179,180]. In rats and primates, the amygdala is associated with the striatum, thalamus, and OFC [181]. The amygdala interacts with the OFC to encode the data that may drive goal-directed behavior [182]. However, according to Winstanley et al., the amygdala has a distinct role in emotional regulation compared to the OFC [183]. Tasks that provoke fear and negative emotions cause the amygdala to become active [184,185]. When asked to make decisions on an inhibition challenge, patients with amygdala injuries exhibit low inhibition. A model in which the processing of negative emotions in BPD is associated with reduced amygdala activation, potentially implying a reduced neural capacity to regulate emotions, has recently challenged the long-held concept of amygdala overreactivity to negative-value stimuli [186,187]. This model is based on meta-analytic evidence [11]. The lack of a link between amygdala volume and impulsivity in most studies could be because the amygdala may play other roles in controlling impulsivity that BIS cannot measure. Romero-Torres et al. suggest an association between hippocampal cannabinoid receptor 1 expression and impulsivity, as well as alcohol seeking and consumption in adolescent male rats [188]. While an overall reduction in amygdala and hippocampal volume has been shown in BPD patients compared to controls, most studies have not found a direct correlation with impulsivity [14,15].

The studies in this review did not discover a connection between WM aberrations and impulsivity in individuals with BPD. Previous work suggests that patients with BPD may have WM dysfunction in cortical areas related to emotional processing and executive functioning [189]. Carassco et al., on the other hand, say that people with BPD have lower FA, which could be because the frontotemporal cortex and orbitofrontal region’s commissural fibers are not as well connected [190]. Further linear correlation models show that patients typically present with increased motor impulsivity and a worsening and intensification of negative affect as the FA value within the fornix decreases. Additionally, without corresponding changes in the FA, it was discovered that participants with BPD had significantly higher RD within the left ATR. As the RD increased within the left ATR, participants demonstrated a decline in attention. Researchers have also linked ADHD to similar alterations in the ATR and WM tracts [34].

In ADHD, only two articles have investigated the relationship between the structural changes of GM and impulsivity [63,64]. Wolfers et al. found no link between GM structural changes and impulsivity [64]. However, Onnink et al. indicated a negative correlation between the volume of the right caudate nucleus and impulsivity. Research demonstrated a connection between the severity of the condition and the caudate reduction, linking a smaller caudate to more symptoms of ADHD, primarily hyperactive/impulsive symptoms [63]. The authors’ hypothesis that structural differences are less noticeable in females than in males is supported by the gender effect and could indicate that men and women have distinct ADHD pathophysiologies [191]. 

In terms of cognitive control, the caudate nucleus is crucial [192,193]. Researchers frequently find structural and functional deficits related to the caudate nucleus in children and adults with ADHD [63,194,195]. According to noteworthy structural MRI research, children with ADHD had a smaller caudate nucleus volume than control children [196]. Task-based fMRI studies have shown that the caudate nucleus is much less active in children with ADHD [197] and adults who had ADHD as children [195] during attention and inhibitory control processes. The finding that individuals with ADHD significantly reduce the volume of WM fiber pathways from the caudate to the parietal lobe suggests that widespread WM underdevelopment in the caudate may play a major role in the persistence of ADHD symptoms, particularly impulsive behavior. Numerous DTI studies conducted on adults and children with ADHD that have shown immature WM organizations involving caudate and cortical structures may also lend support to this theory [132,196,198,199].

Konrad et al. reported an association between impulsivity and FA in the right prefrontal-striatal fiber pathways, which connect the limbic and basal ganglia regions of the brain with the OFC. Some of these results supported what Casey et al. found when they showed a link between impulsivity on a test and FA in prefrontal fiber pathways in ADHD parent-child pairs [88]. Previous research has described impulsivity as a result of compromised inhibitory control functions of the prefrontal-striatal circuit [200]. In this context, it is crucial to remember that a DTI study on women with BPD and concurrent ADHD found a correlation between MD in the inferior frontal WM and other clinical symptoms of BPD, such as dysfunctional affect regulation [201]. An MRI study using a fiber-tracking algorithm found that frontostriatal microstructural properties predicted RT. This correlation was stronger for tasks that needed more control [202]. It is challenging to interpret the authors’ ability to show a positive bilateral correlation between MD and impulsivity in the lingual gyrus.

Significant conclusions were reached by Chiang et al. when investigating WM with the DSI sequence using the mGFA parameter [68]. They proved a negative correlation between mGFA values of the right SLF, which is significant because these regions have been repeatedly cited as dysfunctional in both BPD and ADHD, but neurobiological changes in the SLF have never been correlated with the increase in impulsivity. Furthermore, they proved a negative correlation between the right frontostriatal tract, the DLPFC, and hyperactivity-impulsivity symptoms. This is significant because these regions have been repeatedly cited as dysfunctional in both BPD and ADHD, but neurobiological changes in the SLF have never been correlated with the increase in impulsivity. At the right temporo-occipito-parietal junction, this particular fiber pathway and the cingulum bundle link frontal areas and cortical regions, which are thought to be crucial for processing information pertaining to attentional functions [203]. Some ancillary support for the findings comes from research on patients with intermittent explosive disorder, which demonstrated a connection between impulsive aggression and decreased FA in the SLF [204]. While it is commonly known that the DLPFC is in charge of cognitive control [205,206], a functional MRI study revealed that adolescents with ADHD did not engage these PFC regions during response inhibition trials. In fact, there was less activation in the DLPFC in this group than in HC and BPD during response inhibition trials [207]. This implies that although the impulsivity issues in both disorders are similar, the underlying pathophysiology’s severity and nature differ.

This review and the underlying evidence base have several limitations. We should divide the greatest limitations of this review into two categories: those arising from the limitations of the publications included in it and those arising from the adopted methodology.

First and foremost, the wide scope of this review resulted in the inclusion of studies that demonstrated significant heterogeneity in the groups studied. Different inclusion criteria, such as current comorbidities and pharmacological treatment used, characterize the studies; however, most of them neglect to mention the patients’ psychotherapy history, a crucial factor in both ADHD and BPD. Most of the studies presented did not address the impact of the pharmacotherapy used on the results. Moreover, patients’ treatment histories were based solely on self-reports, which can be prone to errors. It is also clear that various psychiatric comorbidities could have influenced the neurobiological changes described by the authors. Environmental factors such as childhood trauma, substance abuse, and chronic stress can significantly affect both cerebral structure and impulsivity [39,40,208,209,210,211,212]. Most studies in this review did not control for these factors. Examining whether different types of childhood trauma contribute to the distinct clinical profiles of BPD and ADHD is essential. Chronic abuse may lead to prolonged stress hormone exposure, impacting brain regions, such as the hippocampus and amygdala, which are key to emotional regulation, and thus potentially contributing to BPD’s emotional instability [213,214]. On the other hand, acute trauma has the potential to affect areas such as the prefrontal cortex, which is frequently associated with attention and impulse control, thereby aligning with characteristics of ADHD [215,216]. Understanding these differences could clarify how specific trauma patterns shape each disorder and inform more targeted treatments. Future studies should consider these factors so that we can better understand whether the observed neurobiological changes are directly related to ADHD or BPD or are due to external factors. Furthermore, the included studies were characterized by varying methodologies, which were based on techniques used to assess and quantify the volume of gray and white matter in specific brain areas. These techniques are often used in research to study brain structure related to various neurological and psychiatric conditions, as well as to understand normal brain development and aging by comparing the gray matter and white matter alterations between different populations. Due to the different parameters studied, a one-to-one comparison of these studies can be problematic. Inferences drawn from such a comparison may not be conclusive. Researchers may subjectively interpret the results of different methods, which can introduce bias. The theoretical framework or assumptions used in each study may differ, complicating interpretation.

Second, the study populations were primarily adult women, limiting their size. Because of the methodology used, not a single study included adolescents with BPD/ADHD or assessed subgroup differences based on gender, age, race, or ethnicity. Research indicates that gender may influence the symptoms of ADHD and BPD, potentially enhancing intriguing neurobiological correlates that the included studies could not observe. For example, neurobiological changes associated with impulsivity may show variability by gender, probably in relation to the influence of hormones and socialization factors [217]. Including a gender-specific analysis in the study would help uncover whether impulsivity in the disorders studied has a different neurobiological basis in other genders, which could assist in tailoring therapeutic interventions to the individual patient. Studies typically excluded patients with comorbid Axis I disorders, such as mood, anxiety, or substance use disorders, which are common among patients in the study groups. This review mainly covers the adult population of Western countries, which limits the generalizability of the conclusions. Including adolescents would shed new light on the neurobiological changes that occur during adolescence, particularly given the presence or development of both BPD and ADHD symptoms during this period. Additionally, racial heterogeneity could reveal the influence of genetic and cultural factors on neurobiological changes associated with impulsivity, helping clinicians tailor a better approach to ADHD and BPD patients from other demographic groups. Therefore, we cannot assess the generalizability of our results to other populations. Moreover, the studies included in this review are cross-sectional, limiting conclusions regarding causation or the progression of neurobiological variation. Longitudinal research could provide further insight into how brain structures associated with impulsivity develop over time in ADHD and BPD. An understanding of these trajectories could help anticipate outcomes and guide preventive approaches, particularly for adolescents or at-risk populations.

Third, measuring impulsivity was not a straightforward process. The majority of studies examining BPD relied on the BIS-11 scale, focusing primarily on overall impulsivity rather than its individual component traits. Given the nature of the existing literature, this approach was necessary, but future research in this area should be more nuanced. The included articles examining the correlation of impulsivity with neurobiological changes among ADHD patients were even more heterogeneous. They measured impulsivity using four different scales: ASRS, CAADID, DIB-R, and TOVA. Therefore, a study that compares patients with a diagnosis of ADHD or BPD may lack the clarity and research value of a study that compares groups based on assessments of the same scale. Measuring impulsivity may be one factor contributing to the inconsistent results; research indicates that self-report questionnaires do not always correlate with actual behavioral decision-making [218]. While factor analysis research indicates that there are many underlying constructs in the BIS, interpreting the composite score for the BIS is problematic [86]. Additionally, examining impulsivity in terms of its parts (like motor impulsivity and attention impulsivity) instead of a single total score might give us more information about which parts of impulsivity are linked to different parts of the brain in each disorder. Nevertheless, the composite score has been used in the analysis of many articles.

Furthermore, because both disorders often occur together, not screening for ADHD or not excluding people who had ADHD from studies that were included has major flaws because the two conditions share similar neurological and behavioral signs and symptoms. Both BPD and ADHD show abnormalities in brain areas related to impulse control, emotion regulation, and executive functioning, particularly in the prefrontal cortex, amygdala, and striatum. These similarities may confound the findings, as it is difficult to attribute the observed neurobiological changes solely to BPD without considering the potential impact of ADHD. Furthermore, ADHD often co-occurs with BPD, and the presence of both conditions can exacerbate specific symptoms, such as impulsivity and dysregulation of attention, potentially amplifying neurobiological changes. This overlap can lead to variations in brain structures and patterns of connectivity that are not specific to BPD alone. By not controlling for ADHD, studies risk attributing neurobiological changes to BPD that may, in fact, result from or be influenced by ADHD, thus reducing the specificity and reliability of the results. Therefore, it is essential to screen for ADHD and include it as an exclusion criterion during testing to isolate neurobiological markers specific to BPD and ADHD. Given the limitations of the methodology adopted and the availability of literature, it should be mentioned that, due to inconsistent results across different modalities explaining the discrepancy between brain function and structure [219], we did not examine brain function during rest or in task-based studies, which made it difficult to assess impact. In addition, because personality traits are multidimensional, functional MRI, which decodes cognitive processes based on a specific task, would not be suitable for capturing the entire range of personality traits. Studies analyzing the relationship between anatomical and functional changes are necessary.

Head motion is a common challenge in neuroimaging studies, especially with impulsive participants who may struggle to remain still during scans. Even minor movements can introduce significant artifacts, impacting data quality and potentially altering findings. Head motion connected to impulsivity remains a potential confounding factor, underscoring the importance of replication and larger sample sizes to improve reliability in this field [220].

Additionally, the search strategy adopted failed to yield an equal number of articles examining the association of impulsivity with biological changes in ADHD and studies examining patients diagnosed with BPD. This led to an imbalance between the quality and quantity of results in these two disorders. Upon the publication of more papers exploring the correlation between ADHD symptoms and neuroimaging studies, conducting a similar review would be highly beneficial. The methodological limitations of our systematic review also include the potential for publication bias and the restriction of eligibility to studies published exclusively in English. Restricting to English-language publications may introduce linguistic bias, according to methodological studies, although this usually has little effect on effect estimates and conclusions. Limitations that can arise from any systematic review include publication bias and selective reporting of results. Despite our best efforts to include null results, we were unable to include unpublished results, so publication bias is almost inevitable. In an effort to reduce study heterogeneity, we also limited the study populations to those with ICD-10, DSM-3/DSM-4/DSM-5, or later diagnoses. As a result, we did not include some early studies in our systematic review.

Neuroimaging studies, including those analyzing structural alterations in gray and white matter, provide critical insights into the neurobiological underpinnings of impulsivity in ADHD and BPD. However, it is crucial to note that these findings, while advancing our understanding within the context of research, lack validation for everyday clinical use.

The neurostructural underpinnings of impulsivity remain a complex area requiring further investigation, particularly in systematic studies exploring the nuances of ADHD symptomatology. The results of our thorough systematic review give us a new way to examine impulsivity in people with BPD from a neuroanatomical point of view, indicating a different neurostructural basis compared to ADHD. This aligns with a recent, similarly comprehensive systematic review and meta-analysis by Pan et al. examining impulsivity in HC, which partially overlaps with our findings [41].

Further research is necessary to better understand the similarities and differences in the brain-behavior relationships of impulsivity across various diagnostic groups. The biopsychosocial model of BPD and neurobiological studies examining ADHD both emphasize a significant neurobiological component to the clinical expression of these disorders. However, the precise interplay between psychological variables and WM and GM alterations remains unclear. While both frontal and limbic systems are involved in the neural pathways contributing to these disorders, additional research is necessary to elucidate how these systems interact with psychological processing.

## 5. Conclusions

In conclusion, this systematic review emphasizes the distinct neurostructural correlates of impulsivity in BPD and ADHD. While both disorders show impulsivity as one of their main symptoms, the fundamental brain structures associated with this trait are distinct. BPD is primarily associated with abnormalities in the prefrontal cortex and limbic system, whereas the changes observed in ADHD focus on the caudate nucleus and frontostriatal pathways. These findings highlight the complexity of the neurobiological underpinnings of impulsivity and suggest that, despite overlapping symptoms, BPD and ADHD may involve different neural mechanisms. Further research is needed to clarify these differences and their implications for treatment. While neuroimaging advances our understanding of the structural correlates of impulsivity in ADHD and BPD, these findings remain primarily research tools and are not yet applicable for clinical diagnostics. Further studies are required to validate these neurobiological markers before they can reliably inform clinical practice. Research settings limit the application of neuroimaging markers, as further investigation into their reliability and reproducibility is necessary before they can inform clinical diagnostics or treatment. Future studies should examine differences based on gender, race, and the environment to determine if the changes seen in the brain are directly linked to ADHD or BPD, or if they are caused by other factors. This will help us better understand the neurobiological basis of impulsivity and allow us to treat each patient more individually. Additionally, it would be beneficial to include functional MRI (fMRI) results in future studies so that we can see how the brains of people with BPD and ADHD change over time when they are acting on impulses, which is something that structural studies alone cannot do. Future investigations should prioritize exploring the correlation between intricate symptomatology and specific white matter changes. The successful translation of findings from large-scale research to the individual patient level will be paramount in determining the future of neuroimaging in psychiatry, impacting clinical practice, diagnosis, treatment, and prognosis. Clinicians will be better equipped to understand the underlying mechanisms of mental illness and disorders with a robust foundational understanding of the field.

## Figures and Tables

**Table 1:** Types and biological basis of impulsivity across psychiatric disorders.

Disorder	Type of Impulsivity	Biological Basis	Additional Notes
ADHD	Motor impulsivity, attention difficulties	Frontal–striatal circuits	Impairments in inhibitory control contribute to impulsive actions
Borderline Personality Disorder	Emotional impulsivity, impulsive aggression	OFC, amygdala	Associated with emotional dysregulation, leading to reactive behaviors
Bipolar Disorder (Manic Episodes)	Risk-taking, lack of impulse control	PFC, limbic system	Heightened impulsivity during manic episodes, impacting decision-making
Substance Use Disorders	Reward-seeking impulsivity, compulsive behaviors	OFC, ventral striatum, dopaminergic pathways	Driven by reward circuits, leading to compulsive use and cravings
Antisocial Personality Disorder	Aggression-related impulsivity, lack of empathy	OFC, limbic structures	Impulsivity often linked to aggression and disregard for consequences
Eating Disorders	Compulsive eating, emotion-driven impulsivity	OFC, insula	Impulsive eating often triggered by emotional states

**Table 2:** Table with selected characteristics of included BPD studies (N = 17).

Study	Year	Sample					Brain Imaging Modality	Analysis/ Measures	Measurements of Impulsivity	Results
		N	Females (N)	Age	Comorbidities (N)	Medication (N)				
Schaub et al. (2023) [46]	2023	BPD = 45 HC = 109	35 56	27.51 ± 8.03 30.07± 7.18	No comorbidities	Naive 23, medicated 22	MRI	GMV	BIS-11	Negative relationship between GMV in the left IFG and impulsivity.
Sampedro et al. (2020) [47]	2021	BPD = 61 HC = 19	54 13	32.4 ± 7.96 34 ± 4.98	MDD, ADHD, PTSD	Antidepressant and/or antipsychotic	MRI	GMV, Cth	BIS-11	(1) Impulsivity negatively correlated with GMV at the middle and inferior prefrontal areas in the left hemisphere; (2) impulsivity negatively correlated with Cth in the caudal and middle frontal and precentral areas; (3) positive correlation between impulsivity and GMV of PCC, precuneus, and parahippocampal area.
Depping et al. (2018) [48]	2018	BPD = 17 HC = 22	17 22	28.6 ± 7.3 31.4 ± 11.2	MDD history 5, depressive symptoms 8, anxiety disorder 1, SUD 6, eating disorders 4	2 weeks of stable medication	MRI	LGI	BIS-11	Negative correlation between LGI of the medial orbitofrontal gyrus and of the rostral middle frontal gyrus and impulsivity.
Zhou et al. (2017) [49]	2017	BPD = 30 HC = 32	15 16	22.55 ± 1.50 23.08 ± 1.05	No comorbidities	Not taking antipsychotic drugs	MRI	Cth, hemispheric asymmetry	BIS-11	(1) No significant correlation between total impulsivity scores and left-right hemispheric asymmetry in ACC thickness; (2) no significant correlation between total impulsivity scores and left-right asymmetry of AI was found; (3) positive correlation between asymmetry in cortical thickness of ACC and the score of the BIS attention subscale; (4) positive correlation between and left-right hemispheric asymmetries in gray matter volume of AI and the score of the BIS attention subscale.
Depping et al. (2016) [50]	2016	BPD = 17 HC = 22	17 22	28.6 ± 7.3 31.4 ± 11.2	MDD history 5, depressive symptoms 8, anxiety disorder 1, SUD 6, eating disorders 4	2 weeks of stable medication	MRI	GMV	BIS-11	Negative correlation between GMV of the hippocampus, parahippocampus, amygdala, and impulsivity.
Kuhlmann et al. (2013) [51]	2013	BPD = 30 HC = 33	30 33	23.7 ± 4.6 24.4 ± 4.1	MDD 20, PTSD 9, SUD 8, anxiety disorders 10, eating disorders 9, adjustment disorder 1, other personality disorders 16	Unmedicated	MRI	GMV	BIS-11	No correlation between GMV of the hippocampus, amygdala, anterior cingulate cortex, hypothalamus, and impulsivity.
O’Neill et al. (2013) [52]	2013	BPD = 20 HC = 21	20 21	32.6 ± 10.1 30.1 ± 8	MDD, other excluded	All with history of psychotropic medications	MRI	Hippocampus and caudate nucleus volume	BIS-11	(1) No significant correlation between hippocampal volumes and impulsivity; (2)negative correlation between right caudate volume and impulsivity.
Sala et al. (2011) [53]	2011	BPD = 15 HC = 15	11 11	32.8 ± 7.6 34.2 ± 8.1	MDD 8, anorexia nervosa 2, dysthymia 4	Naive 3, antidepressants 4, mood stabilizer 2, antipsychotic 1, mixed medications 5	MRI	GMV	BIS-11	Negative correlation between bilateral DLPFC GMV and impulsivity.
Völlm et al. (2009) [54]	2009	BPD = 7 HC = 6	0 0	35.1 ± 5.8 33.0 ± 8.29	No comorbidities	No current medication	MRI	GMV	IVE	Negative correlations between GMV in the OFC, middle frontal gyrus, precentral and postcentral gyrus, temporal pole, and the inferior and superior parietal cortex and impulsivity.
Soloff et al. (2008) [55]	2008	BPD = 34 HC = 30	22 19	27.5 ± 8 25.6 ± 7.7	No Axis 1 comorbidities	No current medication	MRI	GMC	BIS-11	No correlation between GMC in any examined region and impulsivity.
Zetzsche et al. (2007) [56]	2007	BPD = 25 HC = 25	25 25	26.1 ± 7.1 27.2 ± 6.3	MDD 17, dysthymia 9, panic disorder 9, agoraphobia 5, other anxiety disorders 8, PTSD 8, bulimia 7, somatoform disorders 5, acute psychotic disorder 4	Patients receiving current 20, history of medication 19	MRI	GMV	BIS-11	No correlation between hippocampal GMV and impulsivity.
Hazlett et al. (2005) [57]	2005	BPD = 50 HC = 50	23 20	31.50 ± 9.9 33.2 ± 8.5	other PD 40, SUD 14, MDD history 38	6 weeks medication wash-out period before MRI	MRI	WMV, GMV	BIS-11	(1) Negative correlation between left GMV in BA 25 and impulsivity; (2) negative correlation between right WMV in BA23 and impulsivity; (3) negative correlation between left GMV in BA 10 and impulsivity; (4) positive correlation between left and right WMV in BA 44 and impulsivity; (5) positive correlation between WMV in BA 47 and impulsivity and irritability-assaultiveness.
Quattrini et al. (2019) [58]	2019	BPD = 15 HC = 14	7 4	37.3 ± 8.9 35.6 ± 7.2	MDD with psychotic features, schizophrenia, schizoaffective disorder and substance or alcohol abuse in the last 3 months were excluded	Neuroleptic *n = 10*, SSRI *n = 5*, BDZ *n = 9*, stabilizers *n = 6*	DTI	FA, RD	BIS-11	No correlation between RD and FA and impulsivity.
Lischke et al. (2017) [59]	2017	BPD = 21 HC = 20	21 20	26.21 ± 6.12 26.81 ± 4.89	substance dependance, BD, schizoaffective disorder, schizophrenia or schizotypal personality disorder were excluded	Not taking antipsychotic drugs	DTI	FA, MD	BIS-11, ASRS	No correlation between FA or MD in any region of the CC and impulsivity.
Salvador et al. (2016) [60]	2016	BPD = 43 HC = 43	43 43	32.55 ± 7.32 32.40 ± 11.8	no data	Patients were receiving medications	DTI	FA, MD, GBC, AlFF	DIB-R	No correlation between FA and MD and impulsivity.
Gan et al. (2016) [61]	2016	BPD = 30 HC = 31	14 17	22.10 ± 1.31 22.38 ± 1.62	no comorbidities	Drug-naive	DTI	FA, RD	BIS-11	(1) Negative correlation between FA values for the genu of the CC with unplanned impulsivity; (2) positive correlation between RD values for the anterior thalamic radiation with attention impulsivity; (3) negative correlation between the FA fiber bundles passing through the fornix with positive intensity and motor-impulsivity; (4) after the multiple comparison correction, no correlation between MD and FA and impulsivity.
New et al. (2013) [62]	2013	BPD = 24 HC = 19	5 6	32.0 ± 9.0 28.6 ± 6.9	MDD 24, dysthymia 1, OCD 2, social phobia 4, PTSD 12	No current medication	DTI	FA	BIS-11	No correlations between FA and impulsivity

**Table 3:** Table with selected characteristics of included ADHD studies (N = 6).

Study	Year	Participants					Brain Imaging Modality	Analysis/Measures	Measurements of Impulsivity	Results
		N	Females (N)	Age	Comorbidities (N)	Medication				
Onnink et al.(2014) [63]	2014	ADHD = 119 HC = 107	73 62	36.29 ± 10.90 36.92 ± 11.54	MDD 55, BD 8, anxiety disorder 27, SUD 22, BPD 10, ASPD 3	Naive 16, stimulant 82, atomoxetine 8, medication in past 13	MRI	volumes of caudate	ASRS	(1) Negative correlation between right caudate volume and impulsivity; (2) structural differences are less pronounced in females than in males.
Wolfers et al. (2017) [64]	2017	ADHD = 87 HC = 93	56 64	32.9 ± 9.5 35.1 ± 11.7	1.31 ± 1.3	Psychostimulants or atomoxetine, no other	MRI, DTI	FA, MD, GMV, Cth	ASRS	No correlation between FA, MD, GMV, Cth, and impulsivity in the ADHD group.
Luo et al. (2020) [65]	2020	ADHD = 35 HC = 46	5 5	24.60 ± 2.1 24.24 ± 2.3	No comorbidities	48 h medication wash-out period before MRI	DTI	FA	CAADID	Negative correlation between FA of the left caudate–parietal WM fiber tract and impulsivity.
Onnink et al. (2015) [66]	2015	ADHD = 107 HC = 109	66 62	35.00 ± 10.30 36.08 ± 10.97	MDD 52, anxiety disorder 22, SUD 21, BPD 10	Naive 20, stimulant 64, atomoxetine 9, history of medication 14	DTI	FA, MD, RD, AD	ASRS	No correlation between FA or with MD and impulsivity.
Konrad et al. (2010) [67]	2010	ADHD = 37 HC = 34	16 18	32.5 ± 10.3 30.2 ± 8.2	No comorbidities	Drug-naive	DTI	FA, MD	TOVA	(1) Negative correlation between UF and ATR FA, and impulsivity; (2) positive correlation between MD bilaterally in the lingual gyrus and impulsivity.
Chiang et al. (2022) [68]	2022	ADHD = 68 HC = 84	29 36	28.70 ± 7.85 28.39 ± 7.9	No comorbidities	Drug-naive	DSI	mGFA,	ASRS	Negative correlation between mGFA values of the right SLF and the right frontostriatal tract from the DLPFC and hyperactivity-impulsivity symptoms.

## Data Availability

Data sharing is not applicable.

## Supplementary Materials

The following supporting information can be downloaded at: https://www.mdpi.com/article/10.3390/jcm13226906/s1. Table S1. Risk of Bias Assessment. Table S2. Table with selected characteristics of included BPD studies (N = 17). Table S3. Table with selected characteristics of included ADHD studies (N = 6).
