# Supplementary material for: Impulsivity in ADHD and Borderline Personality Disorder: A Systematic Review of Gray and White Matter Variations
Source: J Clin Med. 2024 Nov 16;13(22):6906. doi: 10.3390/jcm13226906 (PMC11594719; doi:10.3390/jcm13226906)
Supplement: Supplementary file 1 [file jcm-13-06906-s001.zip › jcm-3283801-supplementary.pdf]

Supplement

Systematic Review

Impulsivity in ADHD and Borderline Personality Disorder: A Systematic Review of Gray and White Matter Variations

Łukasz Franczak [1], Piotr Podwalski [1,*], Patryk Wysocki [1], Bartosz Dawidowski [1], Adam Jędrzejewski [2], Marcin Jabłoński [1] and Jerzy Samochowiec [1]

[1] Department of Psychiatry, Pomeranian Medical University, Broniewskiego 26 Street, 71-460 Szczecin, Poland; lukasz.franczak@pum.edu.pl (Ł.F.); patryk.wysocki@pum.edu.pl (P.W.); bartosz.dawidowkski@pum.edu.pl (B.D.); marcin.jablonski@pum.edu.pl (M.J.); jerzy.samochowiec@pum.edu.pl (J.S.)

[2] Independent Clinical Psychology Unit, Pomeranian Medical University, Broniewskiego 26 Street, 71-460 Szczecin, Poland; adam.jedrzejewski@pum.edu.pl

* Correspondence: piotr.podwalski@pum.edu.pl

1. Complete search strategy

SCOPUS:

((("attention deficit hyperactivity disorder" OR ADHD OR "Attention Deficit Disorder with Hyperactivity") OR ("borderline personality disorder" OR BPD OR "Borderline Personality Disorder")) AND ((impulsiv* OR "Impulsive Behavior" OR "Dangerous Behavior") OR ((emotion* OR mood) AND labil*)) AND (("diffusion tensor imaging" OR DTI OR "Diffusion Tensor Imaging") OR ("magnetic resonance imaging" OR MRI OR "Magnetic Resonance Imaging")) AND ("white matter" OR "White Matter" OR "gray matter" OR "Gray Matter"))

EMBASE:

('adhd'/exp OR 'adhd' OR 'attention deficit hyperactivity disorder'/exp OR 'attention deficit hyperactivity disorder' OR 'borderline personality disorder'/exp OR 'borderline personality disorder' OR 'bpd' OR 'borderline state'/exp OR 'borderline state') AND ('impulsiv*' OR 'impulsiveness'/exp OR 'impulsiveness' OR 'violence'/exp OR 'violence' OR (('emotion*' OR 'mood'/exp OR 'mood') AND 'labil*')) AND ('dti' OR 'diffusion tensor imaging'/exp OR 'diffusion tensor imaging' OR 'magnetic resonance imaging'/exp OR 'magnetic resonance imaging' OR 'mri'/exp OR 'mri' OR 'nuclear magnetic resonance imaging'/exp OR 'nuclear magnetic resonance imaging') AND ('white matter'/exp OR 'white matter' OR 'gray matter'/exp OR 'gray matter')

PUBMED:

((("attention deficit hyperactivity disorder" OR ADHD OR "Attention Deficit Disorder with Hyperactivity"[Mesh]) OR ("borderline personality disorder" OR BPD OR "Borderline Personality Disorder"[Mesh])) AND ((impulsivity OR impulsiveness OR"Impulsive Behavior"[Mesh] OR "Dangerous Behavior"[Mesh]) OR ((emotional OR emotion OR mood) AND (unstability or lability)) AND (("diffusion tensor imaging" OR DTI OR "Diffusion Tensor Imaging"[Mesh]) OR ("magnetic resonance imaging" OR MRI OR "Magnetic Resonance Imaging"[Mesh])) AND ("white matter" OR "White Matter"[Mesh] OR "gray matter" OR "Gray Matter"[Mesh]))

2. Abbreviations

ADHD Rating Scale-IV (ASRS), amplitude of low-frequency fluctuations (ALFF), anterior cingulate gyrus (ACC), anterior insula (AI), anterior thalamic radiation (ATR), attention deficit hyperactivity disorder (ADHD), autism apectrum disorder (ASD), axial diffusivity (AD), Barratt Impulsiveness Scale (BIS-11), borderline personality disorder (BPD), corpus callosum (CC), cortical thickness (Cth), diffusion spectrum imaging (DSI), diffusion tensor imaging (DTI), dorsal frontal cortex (DFC), dorsolateral prefrontal cortex (DLPFC), Eysenck's Impulsivity Inventory (IVE), global brain connectivity (GBC), fractional anisotropy (FA), gray matter volume (GMV), healthy controls (HC), inferior frontal gyrus (IFG), inferior fronto-occipital fasciculus (IFOF), mean diffusivity (MD), mean generalized fractional anisotropy (mGFA), middle frontal gyrus (MFG), middle occipital gyrus (MOG), middle temporal gyrus (MTG), obsessive-compulsive disorder (OCD), orbitofrontal cortex (OFC), posterior cingulate gyrus (PCG), prefrontal cortex (PFC), radial diffusivity (RD), Revised Diagnostic Interview for Borderlines (DIB-R), Test of Variables of Attention (TOVA), uncinate fasciculus (UF), white matter volume (WMV)

Supplementary Table S1. Risk of Bias Assessment

Study No.	Schaub et al., 2023	Quattrini et al. (2019)	Chiang et al., 2022	Sampedro et al., 2021	Luo et al., 2020	Hazlett et al., 2005	Depping et al., 2018	Zhou et al., 2017	Lischke et al., 2017	Depping et al., 2016	Onnink et al., 2015	Onnink et al., 2014	Kuhlmann et al., 201	Sala et al., 2011	Konrad et al., 2010	VÖLLM et al., 2009	Soloff et al., 2008	Zetzsche et al., 2007	Salvador et al., 2016	Wolfers et al., 2017	Gan et al., 2016	New et al., 2013	O'Neill et al., 2013
A. Selection Bias - Are individuals selected to participate likely to be representative of the target population? (1=Yes, 0=No)	1	1	1	1	1	1	0	1	0	0	1	1	0	1	1	0	1	0	0	1	1	1	0
B1. Study Design - Sample size ≥ 20 or derived from a power calculation? (1=Yes,	1	0	1	1	1	1	0	1	1	0	1	1	1	0	1	0	1	1	1	1	1	1	1
B2. Study Design - Are inclusion/exclusion criteria clearly described? (1=Yes, 0=No)	1	1	1	1	1	1	1	1	1	1	1	1	1	1	1	1	1	1	1	1	1	1	1
B3. Study Design - Were details of sample characteristics included?	1	1	1	1	1	1	1	1	1	1	1	1	1	1	1	1	1	1	1	1	1	1	1
C1. Insight into social cognition measure - Has the task been validated in any populations? (1=Yes, 0=No)	1	1	1	1	1	1	1	1	1	1	1	1	1	1	1	1	1	1	1	1	1	1	1
C2. Insight into social cognition measure - Has the task been adapted/validated for the population studied? (1=Yes, 0=No)	1	1	1	1	1	1	1	1	1	1	1	1	1	1	1	1	1	1	1	1	1	1	1
D1. Neuroimaging methodology - Is a validated method of data acquisition used? (1=Yes, 0=No)	1	1	1	1	1	1	1	1	1	1	1	1	1	1	1	1	1	1	1	1	1	1	1
D2. Neuroimaging methodology - Is a validated method of data processing used? (1=Yes, 0=No)	1	1	1	1	1	1	0	0	1	1	1	1	1	1	1	1	1	1	1	1	1	1	1
E1. Analysis - Was analysis hypothesis driven? (1=Yes, 0=No)	1	1	0	1	1	0	1	1	0	1	1	1	1	0	1	1	1	1	0	1	0	1	1
E2. Analysis - Has the study controlled for potential confounders? (1=Yes, 0.5=Partial, 0=No)	1	1	1	1	1	1	1	1	1	1	1	1	1	1	1	1	1	1	1	1	1	1	1
E3. Analysis - Has the study adjusted for multiple comparisons if appropriate? (1=Yes, 0=No)	1	1	1	1	1	1	1	1	1	1	1	1	1	1	1	1	1	1	1	1	1	1	1
E4. Analysis - Are statistical methods appropriate for study design/sample size? (1=Yes, 0=No)	1	1	1	1	1	1	1	1	1	1	1	1	1	1	1	1	1	1	1	1	1	1	1
Total score	12	11	11	12	12	11	9	11	10	10	12	12	11	10	12	10	12	11	10	12	11	12	11
Percentage	High	Moderate	Moderate	High	High	Moderate	Low	Moderate	Moderate	Moderate	High	High	Moderate	Moderate	High	Moderate	High	Moderate	Moderate	High	Moderate	High	Moderate

Supplementary Table S2. Table with selected characteristics of included BPD studies (N = 17)

Study	Year	Participants					Control participants			Diagnostic Criteria	Brain imaging modality	Analysis/ measures	Measurements of impulsivity	Measures	Results
		N	Females (N)	Age	Comorbidities	Medication	N	Females (N)	Age						
Schaub et al.	2023	45	35	27,51 ± 8.03	no comorbidities	n=23 naive, n=22 on medication	109	56	30,07± 7,18	DSM-5, SCID I and II	MRI	GMV	BIS-11	BIS-11, BDI-II,	negative relationship between GMV in the left IFG and impulsivity
Sampedro et al.	2021	61	54	32.4 ± 7,96	MDD, ADHD, PTSD	antidepressant and/or antipsychotic	19	13	34 ± 4,98	DSM-IV, SCID-II, DIB-R	MRI	GMV, Cth	BIS-11	BIS-11, BSL, DERS	1) impulsivity negatively correlated with GMV at the middle and inferior prefrontal areas in the left hemisphere 2) impulsivity negatively correlated with Cth in the caudal and middle frontal and precentral areas. 3) positive correlation between impulsivity and GMV of PCC, precuneus and parahippocampal area.
Depping et al.	2018	17	17	28,6 ± 7,3	MDD history n=5 , depressive symtopms n=8,anxiety disorder n=1 SUD n=6 eating disorders n=4	2 weeks of stable medication	22	22	31,4 ± 11,2	DSM-IV	MRI	LGI	BIS-11	BSL-23, BIS-11, HAMD	negative correlation between LGI of the medial orbitofrontal gyrus and of the rostral middle frontal gyrus and impulsivity
Zhou et al.	2017	30	15	22.55 ± 1.50	no comorbidities	not taking antipsychotic drugs	32	16	23.08 ± 1.05	DSM-IV, SCID-II/P	MRI	Cth, hemispheric asymetry	BIS-11	CES-D, BIS-11, SAIS, CTQ, ASQ, PDQ	1) no significant correlation between total impulsivity scores and left-right hemispheric asymmetry in ACC thickness 2) No significant correlation between total impulsivity scores and left-right asymmetry of AI was found 3) positive correlation between asymmetry in cortical thickness of ACC and score of BIS attention subscale 4) positive correlation between and left-right hemispheric asymmetries in gray matter volume of AI and score of BIS attention subscale
Depping et al.	2016	17	17	28,6 ± 7,3	MDD history n=5, depressive symtopms n=8 ,anxiety disorder n=1, SUD n=6, eating disorders n=4	2 weeks of stable medication	22	22	31,4 ± 11,2	DSM-IV	MRI	GMV	BIS-11	BSL-23, BIS-11, HAMD, BDI	negative correlation between GMV of hippocampus, parahippocampus, amygdala and impusivity
Kuhlmann et al.	2013	30	30	23.7 ± 4.6	substance abuse n=8; anxiety disorders n=10; eating disorders n=9;	unmedicated	33	33	24.4 ± 4.1	DSM-IV, SCID-I	MRI	GMV	BIS-11	BIS-11, BDI, STAXI, FAF, CTQ	no correlation between GMV of the hippocampus, amygdala, anterior cingulate cortex, hypothalamus, and implsivity
O'Neill et al.	2013	20	20	32.6 ± 10.1	MDD, other excluded	all with history of psychotropic medications	21	21	30.1 ± 8	DSM-IV	MRI	hippocampus and caudate nucleus volume	BIS-11	BSL-23, BIS-11, HAMD, BDI, EPQ-R, SSS	1) no significant correlation between hippocampal volumes and impulsivity 3) negative correlation between right caudate volume and impulsivity
Sala et al.	2011	15	11	32.8 ± 7.6	MDD n=8, anorexia nervosa n=2, dysthymia n=4	n=3 naive, n=4 antidepressants, n=2 mood stabilizer, n=1 antipsychotic, n=5 mixed medications	15	11	34.2 ± 8.1	DSM-IV, SCID-II	MRI	GMV	BIS-11	DIB, ZAN-BPD, BDHI, BIS-11,HAMD	negative correlation between bilateral DLPFC GMV and impulsivity
Völlm et al.	2009	7	0	35.1 ± 5.8	no comorbidities	no current medication	6	0	33,0 ± 8.29	DSM-IV, SCID-I and II	MRI	GMV	IVE	IQ Quick Test	negative correlations between GMV in the OFC, middle frontal gyrus, precentral and postcentral gyrus, temporal pole, and inferior, superior parietal cortex and impulsivity
Soloff et al.	2008	34	22	27.5 ± 8	no Axis 1 comorbidities	no current medication	30	19	25.6 ± 7.7	DSM-III-R, IPDE	MRI	GMC	BIS-11	DIB, HAMD, BIS-11, LHA	no correlation between GMC in any examined region and impulsivity
Zetzsche et al.	2007	25	25	26.1 ± 7.1	MDD n=17, systhymia n=9, panic disorder n=9, agoraphobia n=5, other anxiety disorders n=8, PTSD n=8, bulimia n=7, somatoform disorders n=5, acute psychotic disorder n=4	n=20 patients (80%) were receiving current and n=19 (76%) had received previous psychotropic medication	25	25	27.2 ± 6.3	DSM-IV, SCID-I and II	MRI	GMV	BIS-11	DIB, HAMD, LHA, BDHI, BIS-11	no correlation between hippocampal GMV and impulsivity
Hazlett et al.	2005	50	23	31.50 ± 9.9	other PD n=40, SUD n=14, MDD history n=38	6 weeks medication wash-out period before MRI	50	20	33.2 ± 8.5	DSM-III-R	MRI	WMV, GMV	BIS-11	BDHI, BIS-11, ALS, AIM	1) negative correlation between left GMV in BA 25 and impulsivity 2) negative correlation between right WMV in BA23 and impulsivity 3) negative correlation between left GMV in BA 10 and impulsivity 4) positive correlation between left and right WMV in BA 44 and impulsivity 5) positive correlation between WMV in BA 47 and impulsivity and irritability-assaultiveness
Quattrini et al.	2019	15	7	37.3 ± 8.9	MDD with psychotic features, schizophrenia, schizoaffective disorder and substance or alcohol abuse in the last 3 months were excluded	neuroleptic n=10, SSRI n=5, BDZ n=9, stabilizers n=6	14	4	35.6 ± 7.2	DSM-IV, SCID-I and II	DTI	FA, RD	BIS-11	SCL-90-R, PSFS, TAS, STAI-Y, BIS-11	no correlation between RD and FA and impulsivity
Lischke et al.	2017	21	21	26,21 ± 6,12	substance dependance, BD, schizoaffective disorder, schizophrenia or schizotypical personality disorder were excluded	not taking antipsychotic drugs	20	20	26,81 ± 4.89	DSM-IV, SCID-II	DTI	FA, MD	BIS-11, ASRS	BSL-23, BIS-11, ASRS, BDI, STAI-T, STAXI-T, MWT	no correlation between FA or MD in any region of the CC and impulsivity
Salvador et al.	2016	43	43	32.55 ± 7.32	no data	patients were receiving medications	43	43	32.40 ± 11.8	DSM-IV, SCID-II	DTI	FA, MD, GBC, AIFF	DIB-R	BSL-23, DIB-R, WAISS-III	no correlation between FA, MD and impulsivity
Gan et al.	2016	30	14	22.10 ± 1.31	no comorbidities	drug-naive	31	17	22.38 ± 1.62	DSM-IV, SCID-I and II, DIVA, PDQ-4	DTI	FA, RD	BIS-11	SAIDS, BIS-11, STAI, CTQ	1) negative correlation between FA values for the genu of the CC with unplanned impulsivity 2) positive correlation between RD values for the anterior thalamic radiation with attention impulsivity 3) negative correlation between the FA fiber bundles passing through the fornix with positive intensity and motorimpulsivity 4) after the multiple comparison correction, no correlation between MD and FA and impulsivity
New et al.	2013	24	5	32.0 ± 9.0	MDD n=24, dysthymia n=1, OCD n=2, social phobia n=4, PTSD n=12	no current medication	19	6	28.6 ± 6.9	DSM-IV, SCID-I and II	DTI	FA	BIS-11	BDI, BPAQ, ALS, BIS-11, STAXI-T	no correlations between FA and impulsivity

Note: SCID = The Structured Clinical Interview, BDI = Beck's Depression Inventory, BSL = The Borderline Symptom List, DERS = Difficulties in Emotion Regulation Scale, HAMD = Hamilton Depression Rating Scale, CES-D = Center for Epidemiologic Studies Depression Scale, SAIS = Self-Assessment Inclusion Scale, CTQ = Childhood Trauma Questionnaire, ASQ = Ask Suicide-Screening Questions, PDQ-4+ = The Personality Diagnostic Questionnaire, GAF = Global Assessment of Functioning, WAISS = Weiss Functional Impairment Rating Scale, STAXI = State-Trait Anger Expression Inventory, FAF = The Functionality Assessment Flowchart, EPQ-R = Eysenck Personality Questionnaire-revised, SSS = Sensation Seeking Scale, DIB = Diagnostic Interview for Borderlines, ZAN-BPD = Zanarini Rating Scale for Borderline Personality Disorder, BDHI = The Buss-Durkee Hostility Inventory, LHA = The Life History of Aggression, ALS = Affective Lability Scale, AIM = Affect Intensity Measure, SCL-90-R = The Symptom CheckList-90- Revised, PSFS = Patient Specific Functional Scale, TAS = Toronto Alexithymia Scale, STAI = The State-Trait Anxiety Inventory, STAXI = State-Trait Anger Expression Inventory, MWT = Maintenance of Wakefulness Test, SAIDS = Sexual Arousal, Interest, and Drive Scale, BPAQ = Buss and Perry Aggression Questionnaire,

Supplementary Table S3. Table with selected characteristics of included ADHD studies (N = 6).

		Participants					Control participants								
Study	Year	N	Females (N)	Age	Comorbidities	Medication	N	Females (N)	Age	Diagnostic Criteria	Brain imaging modality	Analysis/ measures	Measurements of impulsivity	Measures	Results
Onnink et al.	2014	119	73	36.29 ± 10.90	n=55 MDD, n=8 BD, n=27 anxiety disorder, n=22 SUD, n=10 BPD, n=3 ASPD	n=16 naive, n=82 stimulant, n=8 atomoxetine, n=13 medication in past	107	62	36.92 ± 11.54	DSM-IV-TR, DIVA	MRI	volumes of caudate	ASRS	ASRS, WAISS-III, SCID-I and II	1) negative correlation between right caudate volume and impulsivity 2) structural differences are less pronounced in females than in males
Wolfers et al.	2017	87	56	32.9 ± 9.5	1.31 ± 1.3	psychostimulants or atomoxetine, no other	93	64	35.1 ± 11.7	DSM-IV, SCID-I and II, DIVA	MRI, DTI	FA, MD, GMV, Cth	ASRS	ASRS	no correlation between FA, MD, GMV, Cth and impulsivity in ADHD group
Luo et al.	2020	35	5	24.60 ± 2.1	no comorbidities	48-hour medication wash-out period before MRI	46	5	24.24 ± 2.3	DSM-IV, SCID	DTI	FA	CAADID	CAADID, K-SADS	negative correlation between FA of the left caudate-parietal WM fiber tract and impulsivity
Onnink et al.	2015	107	66	35.00 ± 10.30	n=52 MDD, n=22 anxiety disorder, 21= SUD, 10=BPD	n=20 naive, n=64 stimulant, n=9 atomoxetine, n=14 medication in past	109	62	36.08 ± 10.97	DSM-IV-TR, DIVA	DTI	FA, MD, RD, AD	ASRS	ASRS, WAISS-III, SCID-I and II, SART, Delay Discounting task	no correlation between FA or with MD and impulsivity
Konrad et al.	2010	37	16	32.5 ± 10.3	no comorbidities	drug-naive	34	18	30.2 ± 8.2	DSM-IV, SCID-I and II	DTI	FA, MD	TOVA	WURS-k, BADDS, Y-BOCS, BDI, SPAI, TOVA. LPS. MWT-B, AVLT, MWT-B, TMT-a, TMT-b, WCST	1) negative correlation between UF and ATR FA, and impulsivity 2) positive correlation between MD bilaterally in the lingual gyrus and impulsivity
Chiang et al.	2022	68	29	28.70 ± 7.85	no comorbidities	drug-naive	84	36	28.39 ± 7.90	DSM-IV,	DSI	mGFA,	ASRS	ASRS, WAIS-III,K-SADS-E, SADS-L	negative correlation between mGFA values of right SLF and the right frontostriatal tract from the DLPFC and hyperactivity-impulsivity symptoms.

Notes: K-SAIDS = Kiddie Schedule for Affective Disorders and Schizophrenia, SART = Situation Awareness Rating Technique, WURS = Wender Utah Rating Scale, BADDS = Behaviors & Attitudes Drinking & Driving Scale, Y-BOCS = Yale-Brown Obsessive Compulsive Scale, SPAI = Social Phobia And Anxiety Inventory, LPS = The Life Participation Scale, AVLT = Rey Auditory Verbal Learning Test, TMT = The Trail Making Test, WCST = Wisconsin Card Sorting Test, SADS = Social Avoidance and Distress Scale
